# 360° trabeculotomy ab externo with double access for the treatment of congenital glaucoma

**DOI:** 10.5935/0004-2749.2024-0197

**Published:** 2025-06-24

**Authors:** Flávia S. Villas Boas, Ana Catharina Pinho Costa, Christiane Rolim-de-Moura, Carolina P. B. Gracitelli

**Affiliations:** 1 Departamento de Oftalmologia, Universidade Federal da Bahia, Salvador, BA, Brasil; 2 Departamento de Oftalmologia e Ciências Visuais, Universidade Federal de São Paulo, São Paulo, SP, Brasil

**Keywords:** Glaucoma, Glaucoma/congenital, Trabeculectomy, Intraocular pressure, Ophthalmic solutions, Trabecular meshwork, Child

## Abstract

**Purpose:**

This study aims to describe the technique, feasibility, efficacy, and safety of 360° trabeculotomy ab externo with double access for the treatment of congenital glaucoma.

**Methods:**

This paper provides a detailed description of the 360° trabeculotomy ab externo with double access used to treat pediatric glaucoma. The postoperative outcomes of six eyes from six patients who underwent this procedure for primary and secondary congenital glaucoma are also reported.

**Results:**

Six eyes from six patients were included in this study. The median age of the patients at the time of surgery was 1.25 yr (range: 0.27-5.41 yr). The mean preoperative intraocular pressure was 25 ± 5.87 mmHg (range: 18-35 mmHg). At baseline, the mean number of hypotensive eye drop medications used was 2 ± 0.63. Postoperatively, the mean intraocular pressure decreased to 10 ± 2.20 mmHg (range: 9-14 mmHg), and none of the patients required hypotensive eye drops. The most common postoperative complication was hyphema, observed in one case on the first postoperative day; however, it resolved within 7 days.

**Conclusions:**

The 360° trabeculotomy ab externo with double access is a valuable addition to the surgical options for pediatric glaucoma. This technique facilitates a complete 360° ab externo opening of the trabecular meshwork while enhancing surgical safety.

## INTRODUCTION

Childhood glaucoma is a rare condition encompassing a group of diseases characterized by ocular changes, primarily in the anterior segment and iridocorneal angle, leading to increased intraocular pressure (IOP) and subsequent optic nerve damage in infants and children^(1)^. This condition causes characteristic changes, including increased corneal diameter, corneal opacity, Descemet’s membrane ruptures (Haab’s striae), and increased eyeball volume (buphthalmos)^(2^,^3)^. Depending on the etiology, childhood glaucoma is classified as either primary or secondary, with primary congenital glaucoma being the most common type^(1^,^3)^.

Surgical intervention is the primary treatment for primary congenital glaucoma, with goniotomy and trabeculotomy being the most frequently used techniques^(1^-^7)^. Among these, 360° trabeculotomy has demonstrated superior outcomes compared with traditional trabeculotomy^(1^,^3^,^4)^. In 1995, Beck et al.^(8)^ introduced a modified trabeculotomy technique involving the passage of a Prolene suture through Schlemm’s canal ab externo along its entire circumference. The suture was inserted through an access opening in Schlemm’s canal after creating a scleral flap, circumnavigating the ocular circumference, and exiting through the same opening^(8)^. However, limited access to Schlemm’s canal presents a challenge, complicating the insertion and retrieval of the Prolene suture.

To address this issue, Chin et al.^(9)^ proposed a modification in 2012 involving the creation of a deep scleral flap, similar to that used in deep sclerectomy, to facilitate identification of Schlemm’s canal and the insertion of the Prolene suture. Nonetheless, concerns regarding false passage creation during circumferential trabeculotomy and difficulties in achieving full 360° canalization prompted us to further refine this technique to enhance safety.

In this context, this study aims to present a case series of patients undergoing 360° trabeculotomy ab externo with double access (TAEDA), a technique designed to enhance the safety of suture passage, ensuring complete trabecular meshwork incision and Schlemm’s canal inner wall opening.

## METHODS

### Participants

This study included six patients diagnosed with congenital glaucoma (four with primary congenital glaucoma and two with secondary congenital glaucoma) who required surgical intervention. Patients were recruited from the congenital glaucoma sector of the Federal University of Bahia. The institutional review board of the Federal University of São Paulo (CEP/UNIFESP No. 0519/2017) approved the study methods, and all parents or legal guardians provided written informed consent. This study adhered to the ethical principles outlined in the Declaration of Helsinki for human research.

All patients included in the study had a diagnosis of childhood glaucoma, defined by the presence of at least two of the following criteria: IOP >21 mmHg, optic disc excavation (progressively increased cup-to-disc ratio, cup-to-disc asymmetry ≥0.2 when optic discs were of similar size, or focal rim thinning), corneal changes (Haab’s striae or increased diameter), progressive myopia, or a reproducible visual field defect consistent with glaucomatous optic neuropathy. These diagnostic criteria were established by the latest World Glaucoma Association consensus^(10)^.

The inclusion criteria included cases of primary or secondary childhood glaucoma without angular adhesions detected via biomicroscopy. The exclusion criteria comprised eyes exhibiting iris or corneal adhesions as well as other developmental anomalies or secondary disorders that contraindicated angular procedures.

### Surgical technique

All surgeries were performed under general anesthesia. Before orotracheal intubation, an examination under sedation was conducted. IOP was measured using a Perkins tonometer, and whenever possible, the anterior chamber and fundus were assessed. Additionally, the corneal diameter was recorded.

The surgical steps for 360° TAEDA were as follows:

1. A 7-0 Vicryl or 7-0 silk traction suture through the upper and lower peripheral cornea.
2. A superior peritomy was performed, followed by the creation of two scleral flaps: a superficial flap and a smaller, deeper flap, akin to the approach used in nonpenetrating deep sclerectomy^(11)^. Schlemm’s canal was located and confirmed by observing aqueous humor. The superficial flap measured approximately 4 mm in width and 3 mm in length, potentially extending further in cases with a widened limbus. The deep flap measured about 2 mm in width and 3 mm in length. Similar flaps were created inferiorly.
3. The same procedure was repeated inferiorly.
4. A 5-0 Prolene suture fragment was inserted through Schlemm’s canal on one side of the upper flaps and gently advanced until retrieved through the lower canal opening. This was initially performed on one side and subsequently on the other. The suture tips were rounded by heat application before insertion.
5. A viscoelastic solution was injected to maintain anterior chamber stability throughout the procedure.
6. The upper and lower remnants of the Prolene suture were gently pulled to exert traction, disrupting the trabecular meshwork and inner Schlemm’s canal at 180° on one side. The procedure was then repeated on the contralateral suture, completing a 360° trabeculotomy.
7. The scleral flaps and conjunctiva were closed using 8-0 Vicryl everting sutures. The deep flaps were repositioned in their original locations without suturing, as in classic deep sclerectomy.

Only one eye patient underwent the 360° TAEDA procedure. In cases of bilateral disease, the contralateral eye underwent conventional trabeculotomy in one or two sites. This report focuses solely on eyes treated with 360° TAEDA; however, in cases where trabeculotomy was performed at two sites, the procedure was conducted superiorly and inferiorly.

Eyes exhibiting resistance to suture passage through Schlemm’s canal were excluded from undergoing this surgical technique.

### Follow-up

Postoperatively, all patients were prescribed tobramycin-dexamethasone eye drops four times daily for 7 days, followed by a gradual corticosteroid taper over the subsequent weeks.

Tonometry using a rebound or Goldmann tonometer was attempted 1-2 months postoperatively. A rebound tonometer was preferred to avoid sedation. In some unsedated patients, Perkins tonometry could not be performed. Additionally, all patients underwent biometry approximately 30-60 days postoperatively.

### Statistical analysis

Descriptive analysis was performed to summarize demographic and clinical data. Normally distributed variables were presented as mean ± standard deviation, whereas nonnormally distributed variables were reported as median and interquartile range. Skewness/Kurtosis tests and histograms were used to assess normality. Due to the limited sample size, statistical analysis was restricted to descriptive methods.

All analyses were conducted using Stata, version 13 (StataCorp LP, College Station, Texas). The significance level (α, type I error) was set at 0.05.

## RESULTS

Six patients were enrolled, with a median age of 1.25 yr (range: 0.27-5.41 yr). The patient cohort was 67% male (n=4), with 50% presenting unilateral glaucoma (n=3). Primary congenital glaucoma accounted for 67% (n=4), while glaucoma secondary to congenital cataracts accounted for 33% (n=2). Table 1 summarizes these findings.

**Table 1:** Demographic and clinical data of all patients

Preoperative						Postoperative					
	Age at surgery (months)	Cup-to-disc ratio	Horizontal corneal diameter (mm)	Preoperative IOP (mmHg)	Number of preoperative antiglaucoma medications	Preoperative axial length (mm)	Postoperative IOP (mmHg), 30 days	Postoperative axial length (mm), 30 to 60 days	Last postoperative exam (months)	IOP at the last postoperative exam	Number of postoperative antiglaucoma medications at the last postoperative exam
A.M.S.J.	4.1	0.8	13.5	35	1	21.65	14	22.03	5.2	12	0
P.J.A.O.	4.2	-	13.5	18	2	21.49	09	20.53	11.3	09	0
D.F.S.	65	0.75	12	28	3	-	-	-	11	12	2
M.G.	8.9	0.75	13	22	2	22.87	08	22.27	13	09	0
T.F.	3.3	-	13.5	23	2	20.82	10	20.25	13	09	0
T.A.A.	4.5	0.7	13	24	2	20.77	09	19.22	6	08	0

Preoperative IOP averaged 25 ± 5.87 mmHg (range: 18-35 mmHg). All patients were using at least one class of hypotensive eye drops, with a mean usage of 2 ± 0.63 medications. Postoperative IOP averaged 9.8 ± 1.73 mmHg (range: 9-12 mmHg). At 12-month follow-up, only one patient required two classes of hypotensive eye drops.

The most common complication was hyphema on postoperative day 1 (one case), which resolved in 7 days. No other complications were observed.

**Figure 1:**
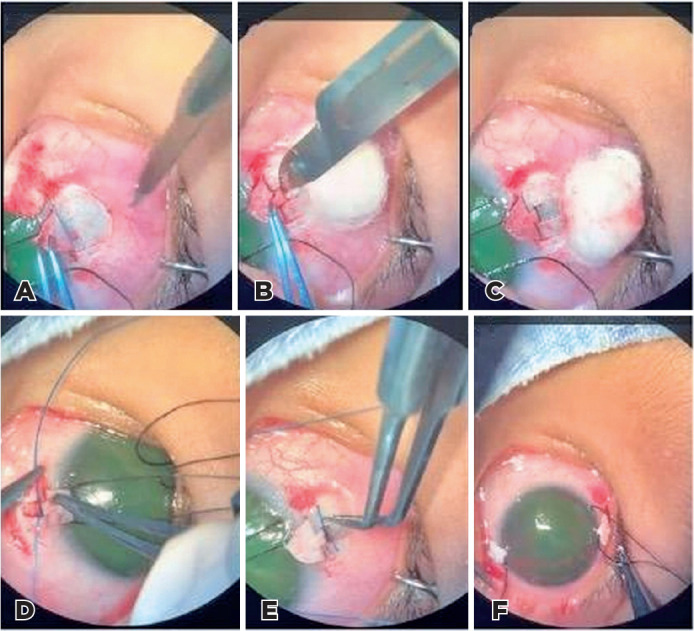
(A) Creation of the superficial scleral flap. (B) Creation of the deep, smaller scleral flap. (C) Identification of Schlemm’s canal. (D) Insertion of a 5-0 Prolene suture on both sides of Schlemm’s canal through the upper flaps. (E) Emergence of the 5-0 Prolene suture fragment through the lower canal opening. (F) Gentle traction applied to the upper and lower Prolene suture remnants, breaking the trabecular meshwork and the inner Schlemm’s canal at 180° on one side

Figure 1 illustrates each step of the procedure.

## DISCUSSION

This study describes a novel technique for childhood glaucoma surgery. To the best of our knowledge, this is the first report utilizing TAEDA for childhood glaucoma, facilitating a 360° trabecular meshwork opening in a single procedure. The technique enables direct visualization of Schlemm’s canal through the creation of a secondary deep flap, similar to the method described by Chin et al.^(9)^, thus ensuring better monitoring of suture progression.

All cases demonstrated effective IOP control postoperatively. The 360° trabecular meshwork opening appears to offer superior IOP reduction compared with more localized trabeculotomy techniques^(5^,^11)^. A recent study comparing conventional and circumferential trabeculotomy reported that the mean IOP over 1 yr was 17.05 ± 5.92 mmHg in the conventional group and 11.0 ± 2.31 mmHg in the circumferential group. After 1 yr, the surgical success rate was 58.44% in the conventional group and 85.71% in the circumferential group^(1)^. Another study evaluating 58 eyes (33 children) after circumferential trabeculotomy and 42 eyes (27 children) after standard trabeculotomy/goniotomy, with a mean follow-up of 7.2 ± 4.0 and 8.2 ± 4.5 yr, respectively, showed a postoperative success (IOP <22 mmHg, with no glaucoma progression or need for additional IOP-lowering surgery) of 81% (47 of 58 eyes) in the circumferential cohort versus 31% (13 of 42 eyes) in the conventional cohort (p<0.0001)^(12)^.

The development of the 360 TAEDA technique was driven by the need for enhanced monitoring during Schlemm’s canal ab externo tunneling. Compared with traditional trabeculotomy, the 360 TAEDA technique offers several advantages: (1) improved safety in locating Schlemm’s canal, as the canal is opened through the deep flap, allowing direct visualization and passage of the Prolene suture; (2) a larger opening area than conventional trabeculotomy, which typically provides an opening of approximately 120°^(9)^. Even if two flaps are created on opposite sides, some areas remain untreated. In contrast, the 360 TAEDA technique ensures a complete 360° opening, confirming canal access via the Prolene suture progression; (3) no need for trabeculotomy probes, which vary in thickness, unlike standardized sutures; (4) the ability to create flaps in opposite locations to access the entire viable trabecular meshwork while avoiding synechiae or areas previously treated with conventional trabeculotomy.

In 2010, Sarkisian Jr.^(13^,^14)^ described the use of an illuminated microcatheter (iTRACK 250A, iScience Interventional, Menlo Park, California) for Schlemm’s canal catheterization in pediatric glaucoma treatment. Compared with Prolene, this method enhances safety by enabling visualization of the microcatheter throughout its path, reducing the risk of creating false passages. However, a potential advantage of the TAEDA technique over illuminated catheterization is its lower cost, as the 5-0 Prolene suture is significantly more affordable than the microcatheter. This cost-effectiveness may increase treatment accessibility in financially constrained settings.

The 360 TAEDA technique modifies the approach described by Beck et al.^(8)^, in which Schlemm’s canal is catheterized circumferentially using Prolene. Beck et al. suggest performing a second flap only if suture progression fails, whereas in the 360 TAEDA technique, a second flap is routinely created. This second flap mirrors the first in configuration, consisting of both a superficial and deep layer. A comparison between the 360 TAEDA technique and a single-flap Schlemm’s canal catheterization with Prolene reveals several advantages: (1) the single-flap, 360° approach is less safe due to a greater area without suture visibility, increasing the risk of suture loss along the path; (2) a single-access Schlemm’s canal catheterization requires an anterior chamber free of synechiae or central adhesions, which might obstruct the passage of the Prolene suture. In cases of corneal opacity, where preoperative ultrasonic biomicroscopy is unavailable, 360 TAEDA is a safer alternative as it does not necessitate suture passage through the anterior chamber’ center; (3) if one end of the Prolene suture fails to progress in the 360 TAEDA technique, conventional trabeculotomy can be performed on that side only. In cases where conventional trabeculotomy was previously performed, the TAEDA technique can be selectively applied to untreated areas, facilitating a full 360° trabecular meshwork opening.

In 2014, Grover et al.^(15)^ introduced gonioscopy--assisted transluminal trabeculotomy (GATT), a technique widely used for glaucoma treatment. GATT involves Schlemm’s canal catheterization and ab interno opening using an intraoperative gonioscopy lens. Compared with GATT, 360 TAEDA may offer advantages in pediatric glaucoma cases, as it enables 360° Prolene suture passage even in eyes with dense corneal opacity. Additionally, it facilitates Schlemm’s canal localization in patients with high iris insertion or anatomical malformations.

Despite its advantages, the 360 TAEDA technique has some limitations. The most notable is the increased surgical time due to the necessity of creating two access points. This is particularly relevant in young children with bilateral glaucoma, where prolonged general anesthesia duration is a concern. Another limitation is the lack of long-term surgical outcome data, given the relatively short follow-up period. However, the benefits of a 360° trabecular meshwork opening for childhood glaucoma treatment are well recognized^(5^,^11)^.

The present study concludes that 360 TAEDA is a valuable addition to the surgical management of pediatric glaucoma, offering a safe and effective method for achieving a 360° ab externo trabecular meshwork opening.
